# Locomotor Activity of *Ixodes ricinus* Females in 900 MHz Electromagnetic Field

**DOI:** 10.3390/life12060884

**Published:** 2022-06-13

**Authors:** Blažena Vargová, Igor Majláth, Juraj Kurimský, Roman Cimbala, Ján Zbojovský, Piotr Tryjanowski, Viktoria Majláthová

**Affiliations:** 1 Center for Applied Research, University of Veterinary Medicine and Pharmacy in Košice, Komenského 73, 040 01 Košice, Slovakia; blazena.vargova@uvlf.sk; 2 Institute of Biology and Ecology, Pavol Jozef Šafárik University in Košice, Šrobárova 2, 041 80 Košice, Slovakia; viktoria.majlathova@upjs.sk; 3 Department of Electrical Power Engineering, Faculty of Electrical Engineering and Informatics, Technical University of Košice, Mäsiarska 74, 041 20 Košice, Slovakia; juraj.kurimsky@tuke.sk (J.K.); roman.cimbala@tuke.sk (R.C.); jan.zbojovsky@tuke.sk (J.Z.); 4 Department of Zoology, Poznań University of Life Sciences, Wojska Polskiego 71C, 60-625 Poznań, Poland; piotr.tryjanowski@gmail.com

**Keywords:** *Ixodes ricinus*, open field, electromagnetic field, tracking, 900 MHz frequency, cell phone, behavior

## Abstract

Mobile telecommunications technologies have become an indispensable part of people’s lives of all ages around the world. They affect personal life and social interactions and are a work tool in the work routine. Network availability requirements and the quality of the Internet connection are constantly increasing, to which telecommunications providers are responding. Humans and wildlife live in the permanent presence of electromagnetic radiation with just a minor knowledge of the impact this radiation has. The aim of our study was to investigate the effect of a 900 MHz electromagnetic field (EMF) on the locomotor behavior of female *Ixodes ricinus* ticks under laboratory conditions. Experiments were performed in the radiation-shielded tube (RST) test and radiation-shielded circular open-field arena placed in an anechoic chamber. Altogether, 480 female *I. ricinus* ticks were tested. In the RST arena, no differences in preference for irradiated and shielded parts of experimental modules were observed; in the open-field arena, the time spent and the trajectory passed was significantly longer in the part exposed to the EMF.

## 1. Introduction

Over the last few decades, cell phone technology has rapidly changed. Nowadays, cell phones represent the core part of human life and cover social interactions and affect the working environment. However, there are limited data on the possible effects of electromagnetic field (EMF) exposure on living organisms. The number of mobile telecommunication devices and duration of usage are increasing at a rapid rate [1]. The number of smartphone users today surpasses three billion worldwide and is predicted to grow by several hundred million in the next few years. Moreover, society claims to no longer be able to live without using technology, which functions by transmitting man-made electromagnetic waves [2]. The Global System for Mobile Communications (GSM), a standard for mobile phones all over the world, operates on a number of carrier frequencies with most networks operating on the 900 MHz frequency. The GSM utilizes a cellular network produced by base station subsystems and provides radio coverage over a wide geographic area, enabling wireless connection of devices [3].

Living organisms are very sensitive to changes in their environment and also react to the presence of or changes in the electromagnetic field. Several authors have studied the impact of an EMF on different types of organisms, ranging from bacteria to invertebrates and vertebrates [4,5,6,7,8,9,10,11]. Currently, it is a known fact that mobile phones affect living organisms [6]. Exposure to an EMF from a mobile phone revealed an impressive decrease (up to 57%) in reproductive capacity and affected the generation time of normal levels in *D. melanogaster* [12,13]. A major field study on insect pollinators provided evidence that electromagnetic smog may have an important ecological and economic impact on pollination that could significantly affect the maintenance of wild plant diversity, crop production and human welfare [14].

*Ixodes ricinus* (Acari: Ixodida) is an important vector of tick-borne pathogens [15]. *Ixodes ricinus* is the most widespread hard tick species in Europe and feeds on a broad range of hosts, serving as reservoirs and vectors of many zoonotic pathogens, including viruses, bacteria and unicellular parasites [16]. The influence of electromagnetic fields as a potential factor that ticks can perceive was studied with the species *Dermacentor reticulatus* and *Hyalomma asiaticum* [17,18,19]. Frątczak et al. [20] found that *I. ricinus* ticks were attracted to the irradiated area of the experimental arena. This effect was significantly stronger for ticks infected with *Rickettsia* spp., suggesting that pathogen presence can alter the ticks’ response to environmental stimuli.

The increasing number of smartphones and their use in nature (cellphones, internet) are changing local environments and affecting living organisms. In this study, we investigated the effect of 900 MHz RF EMF frequency on the locomotor behavior of female *I. ricinus* ticks in the radiation-shielded arena and in the circular open-field arena under laboratory conditions. The 900 MHz RF EMF frequency was chosen a frequency with broad utilization by critical infrastructure operators worldwide.

## 2. Materials and Methods

### 2.1. Model Organism

*Ixodes ricinus* females were used as experimental model organisms. In total, 480 adult female *I. ricinus* ticks were used. Ticks were collected in spring 2018 from vegetation by the flagging method with a white cotton blanket (1 m^2^). Ticks were collected in a forest park in Košice in eastern part of Slovakia (48.745° N; 21.277° E). In the laboratory, all collected ticks were classified at the species level according to Siuda [21] and separated into individual tubes according to developmental stage and sex. After collection, ticks were placed in 50 mL polypropylene tubes with a blade of grass. After transport to laboratory, all collected ticks were kept in polypropylene tubes and were placed in environmental chamber with stable conditions (16 °C and 90% relative air humidity under 16:8 h light–dark regime). Any possible impact of external stimuli, such as odor, that could affect their behavior was minimized. Ticks were kept in environmental chamber for approximately one week and never longer than 1 week. All ticks in polypropylene tubes were placed into the anechoic chamber to acclimatize to the new conditions before starting the experiment with EMF. In this study, we only tested females who are in gengeral feeding on animals and also humans for a longer time, which therefore have an increased probability of transmission of pathogens.

### 2.2. Experimental Setup

#### 2.2.1. Radiation-Shielded Tube Test

A total of 280 female *I. ricinus* ticks were placed into the radiation-shielded tube test (RST). Females had a choice between a shielded and unshielded part. For the detailed description of RST arenas, see Vargová et al. [18].

#### 2.2.2. Source of Radiation for RST

We focused on the artificially emitted electromagnetic field with 900 MHz frequencies. These frequencies represent the range frequency limits that are used in common telecommunications services. The 900 MHz RF EMF frequency was chosen as a frequency with broad utilization by critical infrastructure operators worldwide, and it is used in the Global System for Mobile Communications (GSM). Out of all frequencies used by operators, 900 MHz is the most common.

A signal generator (N5183A, Agilent, MY) was used as a source of RF-EMF connected to the Double-Ridged Waveguide Horn Antenna HF907 (Rohde and Schwarz, Munich, Germany) in anechoic chamber. The output power of the generator was calculated and placed at a distance of 2 m from the antenna with a power flux density of 1 mW/m^2^, which translates to 0.6 V/m in electric field intensity. The temperature in the anechoic chamber was 22 °C, and the humidity was 60%, as measured using a LabQuest 2 unit (Vernier Software & Technology, Beaverton, OR, USA).

#### 2.2.3. Experimental Procedure of RST

In a single RST test, 10 female ticks *I. ricinus* were placed directly in the middle of the test tube. Fourteen tubes were used to conduct irradiation or control sample tests. The total number of females used in the irradiation experiment was 140. The experimental RSTs were placed in the anechoic chamber (model 1710-100, Comtest, Zoeterwoude, The Netherlands). Ticks were exposed to 900 MHz RF-EMF. The control group consisted of 140 ticks; they were not exposed to RF-EMF. The experiment lasted for 24 h. Finally, the total number of ticks in both individual arms of RST was counted and collected. Experiments were performed in the darkness, to prevent the influence of light on behavior.

#### 2.2.4. Radiation-Shielded Circular Open-Field Arena

**Figure 1:**
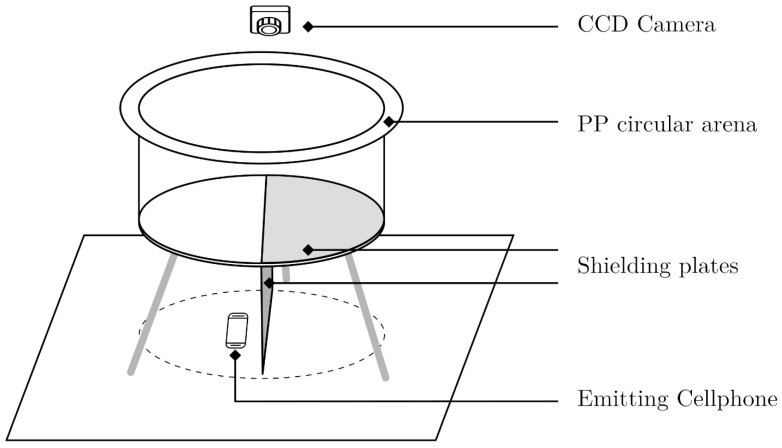
Experimental setup of radiation-shielded circular open-field polypropylene (PP) arena with CCD (charge-coupled device) camera for recording movements of ticks (not to scale).

In specifically modified behavioral arena, 2 zones with different experimental conditions, 1 with EMF and 1 EMF-free, were established (Figure 1). The arena had a circular base with a diameter of 100 cm and a height of 50 cm and was made of white opaque polypropylene (PP), positioned 1 m above the floor. Double-sided 35-micron copper foil on a 1.6 mm epoxy glass laminate desk (DSCED in short) served as the EMF shield. The shield and the source of radiation were rotated randomly between trials in order to avoid bias. The DSCED semicircle attached to the bottom of the arena was perpendicular to the rectangle-shaped shielding plate and divided the space of interest into a shielded/unshielded area. There was no barrier in the arena between the two halves so the ticks could move freely. A cell phone was used as an EMF source. It was placed on the unshielded side, 0.2 m from the center of circular projection to the floor (depicted in Figure 1 as a dashed line).

#### 2.2.5. Source of Radiation for Circular Arena

A common cell phone registered in the cellular telecommunications network was used as a source of RF-EMF. 900 MHz frequency RF-EMF emission was provided by an ongoing call on the cell phone (model Samsung J5 2016, Yongin, Korea). The 900 MHz mode was achieved by changing the phone settings and selecting the network mode 2G only. The phone operation at a maximum power was achieved by limiting the intensity of the GSM base station external signal to the lowest received signal strength. The availability of the external GSM signal was controlled inside the chamber by positioning the electromagnetic shield on the entrance door. The lowest achieved operating intensity of the signal was 0.037 V/m as measured by a signal analyzer (Spectran HF 60105, Aaronia, Augsburg, Germany). Generated by the cell phone during a call, the maximum intensity of EMF in the unshielded part of the arena was 0.61 V/m, while in the shielded one it was 0.09 V/m, both measured in situ by the signal analyzer.

#### 2.2.6. Experimental Procedure of Circular Arena

Ten individual female *I. ricinus* ticks were placed on the centerline of the circular arena. Ticks had a choice between a shielded and unshielded part. The experiment lasted 120 min. After the emission on EMF was stopped, ticks were counted in the shielded and unshielded part and collected in polypropylene tubes with 70% ethanol. In total, 20 experimental trials were performed in the radiation-shielded circular open-field arena, indicating 10 females were used in each trial, which is 200 females altogether. However, digitized movement of 160 individuals was used in the study; 40 females were lost in the field of vision of the camera and thus we were not able to evaluate these tracks. All ticks were used only once in the experiment, in order not to amplify possible individual deviations.

Movement of the ticks in the arena was recorded by a CCD camera (model Panasonic HC-X920, Kanagawa, Japan). The camera was equipped with a wide-angle 29.8 mm lens, and the aperture F1.5 was used. The recording was made in full-HD resolution. Recording rate of 1 frame per second was established experimentally. It provided sufficiently detailed sequences for further tick movement analysis. Up to 12× optical zoom and 5-axis image stabilization were used. Individual experimental observations were recorded and stored separately.

#### 2.2.7. Anechoic Chamber

The experimental arena was placed in the anechoic chamber (model 1710-100, Comtest, Zoeterwounde, The Netherlands), which ensured no external electromagnetic fields affected the experiment. All experiments were performed in an anechoic chamber. Temperature in the anechoic chamber was 22 °C and air humidity was 60%, as measured using a LabQuest 2 unit (Vernier Software & Technology, Beaverton, OR, USA). The interior was illuminated by a pair of low-power EMF free lights placed in two opposite corners of the ceiling of the chamber.

#### 2.2.8. Feature Extraction

We used a camera-based method for analyzing track length and the time spent in the irradiated or shielded part of the arena. The software tool used for analysis of tick movement sequence recordings has been discussed elsewhere. Recorded area was 1920 × 1080 pixels, out of which a circular arena was extracted. Both horizontal and vertical neighbor pixels’ displacement represents approx. 1 mm of distance in real measurement in these directions.

**Figure 2:**
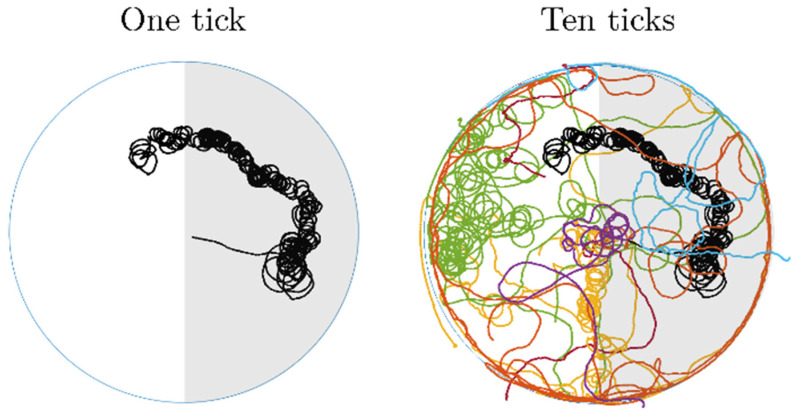
Example of tracking tick movement in the circular arena. Each different color represents track of individual tick movement.

Ticks in the arena were tracked, and digitized information was saved in the data file, i.e., the tick’s identity and x,y coordinates. The coordinates represented the center of the tick’s body (center of the gravity). The tracking performance is depicted in Figure 2. Shadowed area represents the EMF-shielded part of the arena.

The spatio-temporal evidence of ticks inside the arena during the experiment was analyzed. Arena’s digital representation was divided into the shielded and unshielded parts. The analysis consisted of the registration of the tick’s digitized coordinates in the specific part where it belongs. It was performed in a cycle over all digitized coordinates.

Trial time was divided into 10 min periods. Digitized path data of 160 individuals were used to calculate several parameters in these time periods: (1) the total track length (TTL) as the sum of track length contributions walked by ticks included in the experiment; (2) the total track length in the irradiated zone (TTL_I); and (3) the total track length in the shielded zone (TTL_S). Similarly, during the temporal observation, individuals’ presence in the shielded and unshielded parts was performed. Additional parameters were calculated: (4) percentage of the time spent in the arena (PIZ); (5) percentage of the time spent in the irradiated zone (PIZ_I); and (6) in the shielded zone (PIZ_S). The movement dynamics is presented in the form of walked distance per sample (WDS) in (mm/s). It was derived as increment of individual track over the time.

#### 2.2.9. Statistical Analysis

The associations between exposed or shielded preferred zone in RST arenas were tested by the use of Fisher’s exact test. Number of ticks in irradiated and shielded zones was compared. *p* value < 0.05 was considered as significant.

The significance of the preferred zone in the radiation-shielded circular open-field arena was tested by application of Wilcoxon Signed-Rank Test. The total track length in the irradiated zone (TTL_I) and the total track length in the shielded zone (TTL_S) was compared. Similarly, percentage of the time spent in the irradiated zone (PIZ_I) and in the shielded zone (PIZ _S) was calculated. Walked distance per sample (WDS) in the irradiated and shielded parts was also compared. *p* value < 0.05 was considered as significant.

All statistical analyses were performed in SPSS Statistics Software (IBM Company, Chicago, IL, USA).

## 3. Results

### 3.1. Radiation-Shielded Tube Test

**Figure 3:**
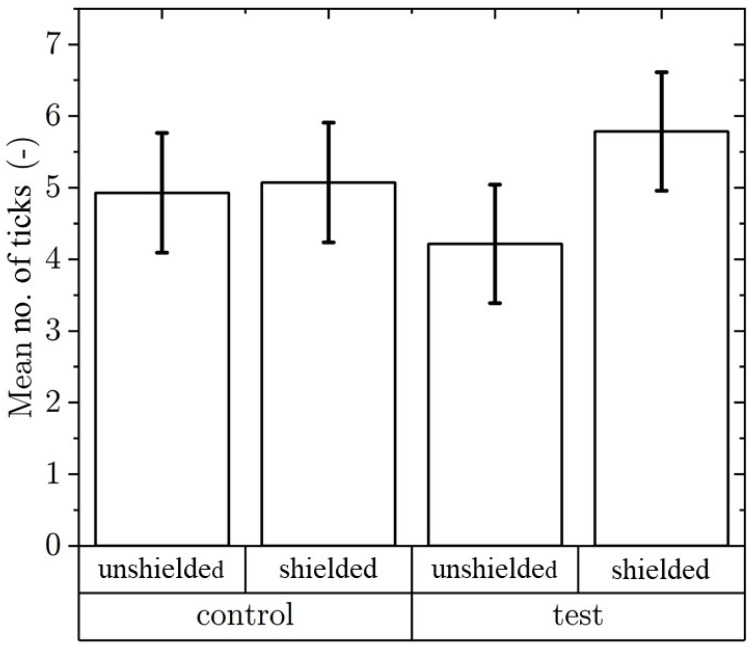
Mean number of *Ixodes ricinus* females per trial in the RST arena in unshielded or shielded parts (n = 140, trials = 14).

In total, we performed tube tests with 280 *I. ricinus* females. When analyzing the impact of 900 MHz, 59 ticks in total moved toward the irradiated part of the tube. That represents 42% of tested ticks, while 81 individuals (58%) reached the shielded part of the tube. In the control group, out of the total number of tested females, 69 (49%) moved toward the unshielded part and 71 (51%) moved toward the shielded part of RSTs. Fisher’s exact test did not show significant differences (*p* = 0.2803) in preference for irradiated parts of experimental modules or any difference when considering the side of the module in the experimental and control groups (*p* = 1) (Figure 3).

### 3.2. Radiation-Shielded Circular Open-Field Arena

**Figure 4:**
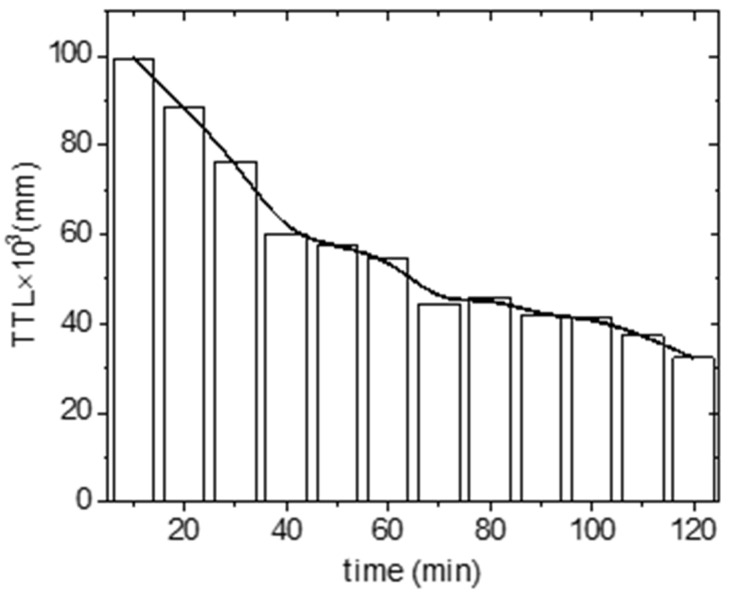
The total traveled length (TTL) by Ixodes ricinus females during the 120 min of the experimental trials. Boxes are sums of single track lengths (n = 160).

After ticks were released into the center of the circular arena, they immediately started to explore the arena. No evidence of escape was observed. Successfully digitized movement of 160 individuals was an input for the statistical file. The locomotor activity was the highest in the first ten minutes, during which the total length traveled was the highest, and then gradually decreased with experimental time, as shown in Figure 4.

**Figure 5:**
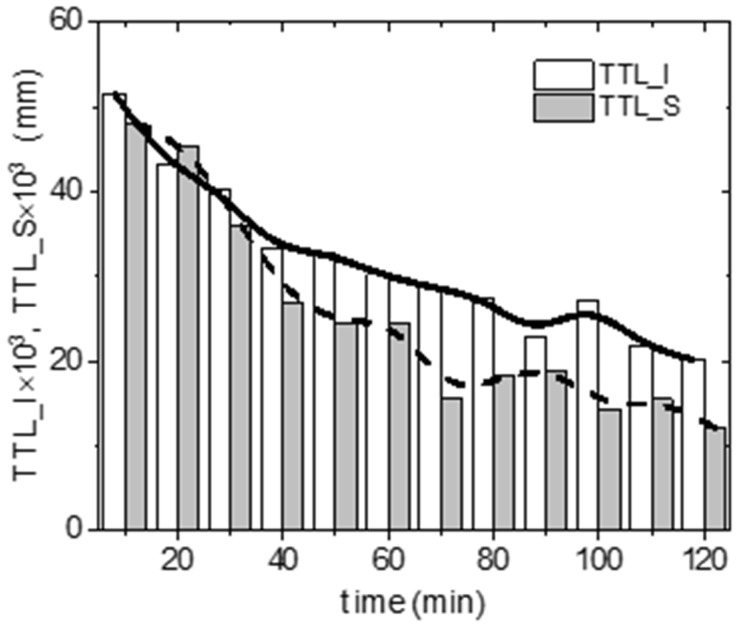
The changes in total traveled length by Ixodes ricinus females in irradiated (TTL_I) or shielded (TTL_S) arena zones during the 120 min of the experimental trials. Boxes are sums of track lengths (n = 160).

When comparing the TTL in the irradiated (TTL_I) and shielded (TTL_S) parts of the arena, we observed similar movement in the first 30 min of the experiment. After this time, the TTL was higher in the irradiated part. After one hour of exposure, the length traveled in the irradiated zone was significantly higher (Figure 5).

**Figure 6:**
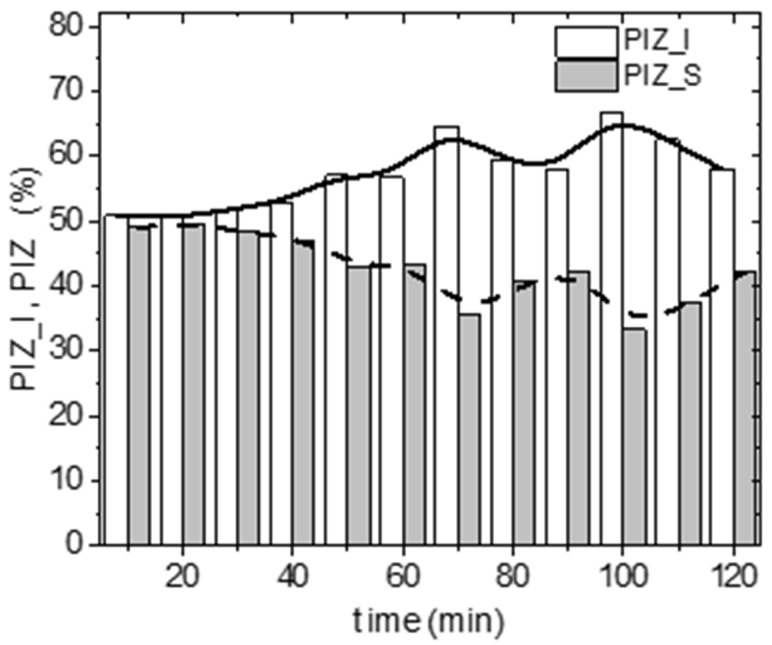
The percentage of time spent by Ixodes ricinus females in irradiated (PIZ_I) or shielded (PIZ_S) arena zones during the 120 min of the experimental trials (n = 160).

Differences were observed in the time spent in specific parts of the arena. The percentage spent in the irradiated part (PIZ_I) ranged from 50.5 to 66.7%, and in the shielded part from 33.3 to 49.5% (Figure 6).

Individuals walked significantly differently when irradiated. It was reflected in the changed movement dynamics of ticks in both irradiated and shielded zones. It is indicated by a median comparison of absolute distance records between the two following positions of the individuals, i.e., walked distance per sample (WDS) in (mm/s), as recorded in both zones and the whole arena. A one-sample Wilcoxon test for 10, 60 and 120 min periods in irradiated and shielded zones was carried out against the median of the WDS parameter calculated for the whole arena in the corresponding periods. The significance level was 0.05 (Table 1).

**Figure 7:**
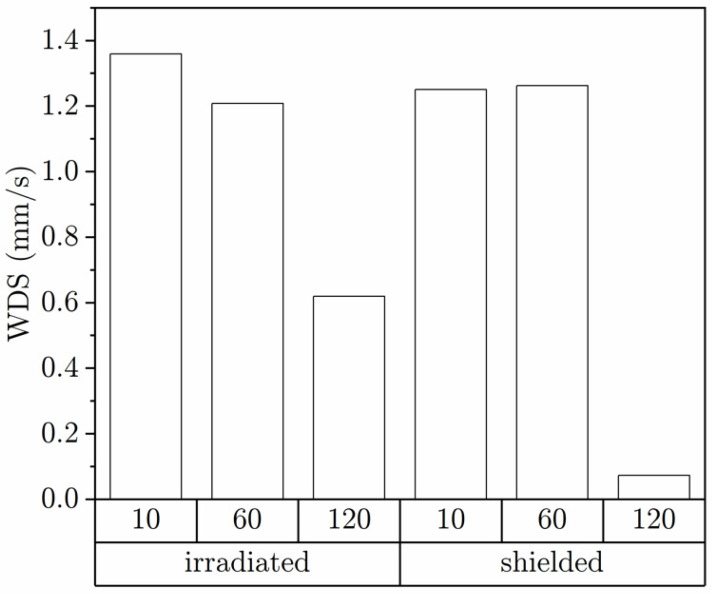
The comparison of change in movement dynamics analyzed during trials.

The observed change in movement dynamics during trials is shown in Figure 7. Analyzed WDS values are presented as columns grouped by the trial duration and the zone shielding disposition, respectively.

## 4. Discussion

The electromagnetic field is a phenomenon that is present in the ecosystem, and all living organisms evolved in the presence of weak omnipresent geomagnetic fields. Over the last few decades, artificial sources of EMFs have expanded rapidly and are affecting all living and nonliving systems [22]. Man-made EMFs, previously unknown for organisms, could interfere and disturb normal physiological functions [10]. The question of whether this disturbance carries information that transforms into a subsequent reaction or causes damage or disorientation is not answered yet. Studying the impact of artificial EMFs on living organisms, as a relatively new phenomenon that has appeared in nature in connection to human activity, is important to clarify how EMFs affect nature and organisms [18,19,20].

In our study, we performed two types of experiments, where *I. ricinus* was a model organism and 900 MHz was the selected frequency. The first type of experiment was performed in RTS modules, a modified T maze for testing the preference for area with an EMF versus a shielded area, with the EMF being emitted by a generator and antenna. In the second type of experiment, the exploratory behavior of ticks was monitored in an open-field arena and the source of the EMF was an ongoing call on the cell phone registered in the GSM network.

The movement toward the source of an artificial EMF in a natural habitat was also observed in wild bees and bee flies [14].

Based on our knowledge, until now, no study has been carried out with a call in progress as the source of a 900 MHz EMF. Our data clearly indicate that *I. ricinus* females moved faster, walked longer distances in the arena and stayed longer in the irradiated zone of the arena in the presence of a 900 MHz frequency EMF originating from the cell phone during a call. Several studies have focused on the walking and questing behavior of ticks in dry versus humid conditions, as air humidity is the key factor for tick activity and survival [23,24]. Few authors have focused on the effect of extremely low EMF frequencies on insects’ walking behavior [25,26,27]. Wyszkovska et al. showed that exposure to relatively high levels of extremely low frequencies of EMFs, generated by overhead power transmission lines, affects the behavior, neuronal and muscular responses and levels of heat shock protein in the desert locust. Extremely low frequency EMFs significantly alter the normal walking behavior of locusts, with the distance traveled being reduced following exposure to an EMF [26]. However, our findings suggest that *I. ricinus* females walk significantly faster. Moreover, our results indicate that ticks walked faster at the beginning of the experiment and gradually walked slower, which may be due to either exploratory behavior, where ticks habituate to a new environment, or they gradually adapted to the presence of the field. Favre and Johansson [28] described that the EMF disturbed the colony of honeybees which produced strong worker pipping signals. Such signals are typically produced shortly before take-off of the swarm, or as a sign of a disturbed colony.

After one hour of a phone call, the traveled distances observed in the irradiated zone were significantly longer. Buczek et al. described the locomotor activity of *D. reticulatus* ticks in their natural environment. Questing *D. reticulatus* females walked a greater distance than males [29]. Wyszkovska et al. [26] discovered that EMFs reduce the distance walked in locusts. Jacskon et al. [30] investigated the effect of the static electric field on the locomotor activity of cockroaches and explained significant changes in locomotion—less distance covered, slowed walking and more frequent turns. We can assume that the 900 MHz frequency used in our experiment had the opposite effect, inducing a longer distance traveled in *I. ricinus* ticks.

## 5. Conclusions

Cell phone use, an exponentially expanding phenomenon introducing electromagnetic load in the environment, inevitably has a biological effect on all living organisms. This does not exclude ticks, epidemiologically very important parasites. EMFs emit a very weak signal to animals (vertebrate and invertebrates), and the evidence of animal response to this signal may be diminished by the plethora of other, stronger signals. To provide evidence unequivocally showing the response to the presence of an EMF is difficult and needs different approaches, such as behavioral tests, and possibly evidence on molecular levels. The number of publications in this field is rising very slowly. In our pilot study, we determined that ticks reacted to the presence of a man-made EMF and a change in the *I. ricinus* female tick locomotor behavior occurred when exposed to 900 MHz frequency. In future studies, it is crucial to test other frequencies, other EMF sources, other tick species, and possibly use the results of behavioral tests as a starting point leading to studying the impact and the changes triggered by EMF exposure at the cellular or molecular level.

## Figures and Tables

**Table 1:** The effects of cell phone radiation on *Ixodes ricinus* females walking dynamics. One-sample Wilcoxon Signed-Rank Test at significance level of 0.05.

Trial Time	WDS Median in Whole Arena	Zone	WDS Median	*p* Value
(min)	(mm/s)		(mm/s)	
10	1.306	Irradiated	1.359	<0.001
		Shielded	1.250	0.384
60	1.232	Irradiated	1.208	0.183
		Shielded	1.262	<0.001
120	0.508	Irradiated	0.619	<0.001
		Shielded	0.073	<0.001

One-sample Wilcoxon Signed-Rank Test at significance level 0.05.
